# Spinal cord imaging in preclinical research

**DOI:** 10.4103/NRR.NRR-D-25-00728

**Published:** 2025-09-03

**Authors:** Lei Cao, Ruiqing Ni

**Affiliations:** Institute for Regenerative Medicine, University of Zurich, Zurich, Switzerland; Institute for Biomedical Engineering, ETH Zurich & University of Zurich, Zurich, Switzerland; Department of Nuclear Medicine, Inselspital University Hospital Bern, Bern, Switzerland

**Figure 1:**
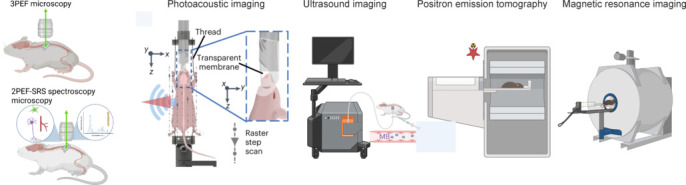
Different approaches for *in vivo* imaging of the spinal cord in small animal models. Created with BioRender.com. 2, 3PEF: Two, three-photon excited fluorescence; MB: microbubble; SRS: stimulated Raman scattering.

The spinal cord links the brain and the peripheral nervous system and has important sensory and motor functions. Impairments in the spinal cord occur in different diseases, such as spinal cord injury, multiple sclerosis, pain, motor neuron diseases, and neurodegenerative diseases. Imaging of the spinal cord has been challenging, partly due to its small size and deep anatomical location. Additionally, in an animal model, motion artifacts further influence the *in vivo* imaging quality of the spinal cord. Recent advances have pushed boundaries for *in vivo* imaging in living animals (even behaving animals). This includes high-resolution optical intravital imaging, Raman spectroscopy, mesoscopic resolution optoacoustic imaging, positron emission tomography (PET), magnetic resonance imaging (MRI), and functional ultrasound imaging (**Figure 1**). In addition, new genetic circuit tracing tools have been combined with *in vivo* imaging tools for small animal imaging. This helps our understanding of the physiology and pathology of the spinal cord at cellular, molecular, and circuit levels. Additionally, *in vivo* imaging of the spinal cord has also enabled the evaluation of the efficacy of both pharmacological and nonpharmacological treatments, and monitoring of the recovery progress. In this perspective, we discuss recent advancements in *in vivo* spinal cord imaging in animal models, as well as challenges and future outlooks.

**Recent technological advances: *Optical imaging:*** Intravital microscopy, such as real-time epifluorescence or time-lapse two-photon laser scanning microscopy, provides high-resolution imaging of the spinal cord in animal models. However, challenges in long-term, motion-stable optical access, imaging depth, and inflammatory responses that could confound the results exist. The spinal cord undergoes rapid, nonrigid movements during breathing, heartbeat, and locomotion. Motion artifacts complicate real-time imaging, especially in awake/behaving animals. Additionally, surgical preparations for spinal cord imaging in animal models often induce inflammation and fibrosis that compromise optical access. Spinal cord inflammation can disrupt the sympathetic nervous system’s regulation of the gastrointestinal-associated immune system, leading to broader physiological consequences, including metabolic alterations and meta-inflammation. Wu et al. (2022) developed minimally invasive intervertebral windows that preserve the ligamentum flavum, enabling repeated two-photon fluorescence and stimulated Raman scattering imaging at subcellular resolution for up to six months without an inflammatory response. This technique allows visualization of multiple tissue components simultaneously, including green fluorescent protein-labeled microglia, blood vessels labeled with Texas Red dextran, collagen (via second harmonic generation), adipose tissue and myelin (via stimulated Raman scattering; Wu et al., 2022). Additionally, Wu et al. (2024) demonstrated that microglia establish direct contact with myelinated axons at nodes of Ranvier in a random scanning pattern under normal conditions. Following axonal injury, microglia rapidly transform this contact into a robust wrapping form via purinergic P2Y12 receptors and prevent acute axonal degeneration from extending beyond the nodes. Moreover, Shekhtmeyster et al. (2023) developed translaminar imaging across spinal cord layers in behaving animals by chronically implanting microprisms and wearable microscopes with custom-compound microlenses. This system addresses common limitations of previous wearable microscopes, such as restricted working distance, resolution, contrast, and achromatic range. Using such a system, Shekhtmeyster et al. (2023) further demonstrated that in behaving mice, dorsal horn astrocytes in the spinal cord showed sensorimotor program-dependent and lamina-specific calcium excitation; in contrast, tachykinin precursor 1-expressing neurons exhibited translaminar activity in response to acute mechanical pain but not locomotion. Additionally, to inhibit fibrotic tissue growth after laminectomy, Ahanonu et al. (2024) used fluoropolymer membranes (Teflon-AF 2400) and demonstrated long-term optical imaging of the spinal cord in awake and behaving mice. These advances in wearable microscopes, with motion-stabilization algorithms, have enabled long-term tracking of cellular dynamics over extended time windows and laminae of the spinal cord, which is critical for longitudinal studies of spinal cord function and pathology in progression and recovery processes. Cheng et al. (2024) developed micrometer-resolution three-photon microscopy, using a 1320-nm excitation source, to image ~550 μm deep *in vivo* of the spinal cord of live mice with implanted chambers. This enabled measuring blood flow speeds, and mapping vasculature, the layer-specific neural degeneration after venule occlusion and tracked perivascular microglia migration in the spinal cord.

***Optoacoustic imaging:*** While optical techniques provide exceptional spatial resolution, they are limited by depth penetration. Complementary molecular imaging approaches have emerged to address this limitation. Compared with conventional optical methods, optoacoustic imaging combines optical excitation with acoustic detection to achieve mesoscopic-resolution imaging at greater depths (several centimeters). Combes et al. (2025) recently employed spiral volumetric optoacoustic tomography (spatial resolution of 60 μm) to investigate changes in the oxygen saturation levels in the spinal cords of M83 transgenic mice with Parkinson’s disease. Unlike manual segmentation, complementary structural MRI data from the same mice were processed via deep learning-based automatic segmentation (spinal cord toolbox) to delineate the white matter and gray matter of the spinal cord. This approach improves accuracy and efficiency, enabling robust quantification of structural alterations (Combes et al., 2025). This study revealed significantly lower oxygen saturation levels in the spinal cords of M83 mice than in those of wild-type controls, suggesting that impaired oxygen metabolism is linked to neurodegeneration.

***Novel PET imaging tracers:*** PET using the synaptic vesicle glycoprotein 2A tracer [^11^C]UCB-J enabled detection of the longitudinal progression of damage and synaptic loss in mouse and rat unilateral spinal cord contusion injury models. This approach has added value compared with the metabolic alterations shown by [^18^F]-fluorodeoxyglucose PET and anatomical MRI. Additionally, van der Weijden et al. (2025) recently reported a new imaging tracer, [^11^C]MeDAS, for directly quantifying myelin loss in the spine of rat models of experimental autoimmune encephalomyelitis, lysophosphatidylcholine, and spinal cord injury, as well as in patients with multiple sclerosis. In addition, hybrid approaches using PET-MRI are used to combine molecular specificity with anatomical precision. This finding highlights the critical need for specific markers of demyelination.

***Functional imaging:*** Functional imaging of the spinal cord, such as using functional ultrasound imaging and MRI, has enabled the visualization of effects in real time and after neuromodulation. Agyeman et al. (2024) developed portable functional ultrasound imaging and adaptive MRI protocols for studying spinal cord function with high spatiotemporal resolutions of 100 µm and 10 ms. The effect of spinal cord electrical stimulation in both humans and animal models can be visualized by the hemodynamic responses detected by functional ultrasound imaging, which is highly relevant for assessing the effect of clinical neuromodulation (Agyeman et al., 2024). The availability of ultrahigh-field MRI (7T–11.7T) in humans has greatly improved the spatial resolution for lesion detection in the spinal cord of patients with multiple sclerosis or spinal cord injury (van der Weijden et al., 2025). Moreover, Cho et al. (2024) reported that targeting the hypothalamic region via deep brain stimulation improved the ability of patients to walk after spinal cord injury through reorganization of residual lumbar-terminating projections from brainstem neurons.

**Challenges in spinal cord imaging in animal models:** In addition to the challenges already solved by the aforementioned advancements, additional challenges still exist in imaging the spinal cords of animal models: quantification and standardization of imaging data across laboratories remain problematic owing to variability in protocols and analysis methods. Diffusion tensor imaging metrics, for example, are sensitive to axonal damage but lack universal cutoff thresholds. Multicenter initiatives aimed at standardizing quantitative MRI protocols for spinal cord injury and degenerative myelopathy in humans are ongoing. A similar standardization for small animal spinal cord imaging is needed. The neuroanatomical-functional paradox, i.e., where similar lesion sizes result in divergent outcomes, is a well-documented phenomenon in the spinal cord. Variable functional eloquence in spinal cord tissue and lesion heterogeneity have posed challenges for replication of the results in small animals and interpretation of results across different experimental protocols. In addition, species differences between rodents and humans and nonhuman primates complicate translational research in spinal cord imaging. Anatomical and functional differences, such as the size and locomotor circuits, exist between the rodent and human spinal cord.

**Future prospects:** Multimodal imaging integration will provide comprehensive mapping of molecular, structural, and functional changes in the spinal cord. Additionally, machine learning approaches are increasingly applied in motion correction, image acquisition, and analysis, such as segmentation and registration. In addition, developing more humanized models, or organoids, may better mimic human spinal cord biology and improve translational relevance. Additionally, understanding the mechanisms underlying the neuroanatomical–functional paradox is essential for interpreting the results and for developing effective therapies. Owing to compensatory plasticity in spared circuits, 3D analysis *in vivo* or *ex vivo*, such as additional tractography analysis or via light sheet microscopy, would be beneficial for tracing axons not relatively close to the lesion epicenter. Advanced imaging techniques that can map functional connectivity and cellular interactions may help resolve this paradox and improve the prediction of outcomes in both preclinical models and clinical settings.

**Figure 2:**
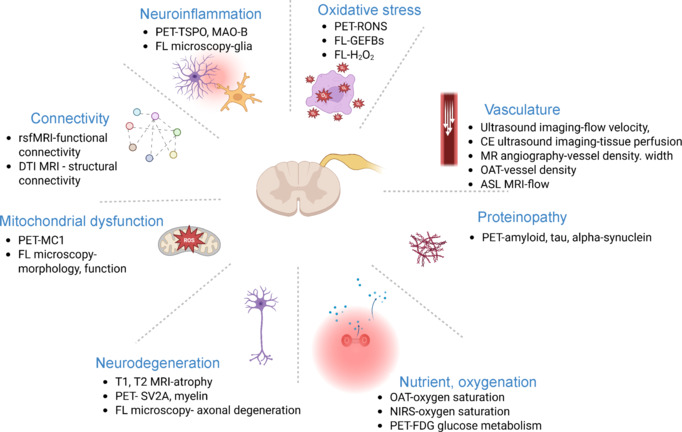
Physiological and pathological parameters detected by *in vivo* imaging in the spinal cords of animal models. Created with BioRender.com. ASL: Arterial spin labelling; CE: contrast enhanced; DTI: diffusion tensor imaging; GEFBs: genetically encoded fluorescent biosensors; FL: fluorescence microscopy (2 photon or 3 photon); MAO-B: monoamine-oxidase B; MC1: mitochondrial complex 1; MR: magnetic resonance; MRI: magnetic resonance imaging; NIRS: near-infrared spectroscopy; OAT: optoacoustic tomography; PET: positron emission tomography; RONS: reactive oxygen and nitrogen species; rsfMRI: resting state functional magnetic resonance imaging; SV2A: synaptic vesicle glycoprotein 2A; TSPO: translocator protein.

Previous single-cell transcriptomics combined with imaging has revealed heterogeneity in spinal cord cell populations and identified 16 sympathetic motor neuron clusters distinguishable by spatial localization and gene expression patterns. This molecular diversity may explain differential vulnerability to disease and injury, with important implications for targeted therapeutic approaches. Advanced *in vivo* imaging techniques have provided real-time insights into the cellular and molecular mechanisms underlying spinal cord function and pathology (**Figure 2**). Recent imaging studies have also revealed the critical role of microglia and astrocytes in spinal cord function and pathology. Tai et al. (2023) demonstrated that targeting mitochondrial metabolism via tricarboxylic acid cycle enzymes in the spinal cord of experimental autoimmune encephalomyelitis mouse model can be used to restore axonal energy deficits. Therefore, further imaging study combined with single-cell analysis and neuron-glia interaction will be warranted.

Moreover, *in vivo* imaging enables the tracking of cellular responses to therapeutic interventions, such as synaptic integrity, myelin and functional connectivity, and provides valuable insights into mechanisms of recovery and potential optimization. Cho et al. (2024) used imaging to demonstrate how hypothalamic deep brain stimulation reorganizes residual spinal projections to improve walking after spinal cord injury, providing a mechanistic basis for this therapeutic approach. *In vivo* imaging could guide the targeting of specific spinal segments or cell populations for neuromodulation or identify patients most likely to benefit from particular interventions on the basis of their imaging profile.

**Conclusions:** Advanced *in vivo* imaging techniques, such as long-term optical translaminar imaging, molecular imaging, and functional/structural imaging techniques in the spinal cord of animal models, have enabled the visualization of complex structures and cellular interactions in preclinical models. These tools not only improve our understanding of spinal cord pathophysiology but also accelerate the discovery of novel therapeutic targets for spinal cord impairments. Future studies should integrate multimodal imaging with advanced image analysis and standardization, and single-cell analysis to improve the translational value of imaging in the spinal cord of animal models.

*This work was supported by SNSF (to RN).*
